# Clinical and pathologic factors associated with survival in young adult patients with fibrolamellar hepatocarcinoma

**DOI:** 10.1186/1471-2407-5-142

**Published:** 2005-10-31

**Authors:** Laura E Moreno-Luna, Oscar Arrieta, Jorge García-Leiva, Braulio Martínez, Aldo Torre, Misael Uribe, Eucario León-Rodríguez

**Affiliations:** 1 Department of Gastroenterology, Instituto Nacional de Ciencias Médicas y Nutrición "Salvador Zubirán" (INCMNSZ), Mexico City, Mexico; 2 Department of Medical Oncology, Instituto Nacional de Cancerología (INCan), Mexico City, Mexico; 3 Universidad Nacional Autonoma de Mexico (UNAM), Mexico City, Mexico; 4 Department of Pathology, Instituto Nacional de Ciencias Médicas y Nutrición "Salvador Zubirán" (INCMNSZ), Mexico City, Mexico; 5 Department Hemato-Oncology, Instituto Nacional de Ciencias Médicas y Nutrición "Salvador Zubirán" (INCMNSZ), Mexico City, Mexico

## Abstract

**Background:**

Fibrolamellar Carcinoma (FLC), a subtype of hepatocellular carcinoma (HCC), is a rare primary hepatic malignancy. Several aspects of the clinic features and epidemiology of FLC remain unclear because most of the literature on FLC consists of case reports and small cases series with limited information on factors that affect survival.

**Methods:**

We did a retrospective analysis of the clinical and histological characteristics of FLC. We also determined the rate of cellular proliferation in biopsies of these tumors. We assessed whether these variables were associated with survival.

**Results:**

We found 15 patients with FLC out of 174 patients with HCC (8.6%). Between patients with these neoplasms, we found statistically significant survival, age at onset, level of alpha fetoprotein, and an earlier stage of the disease. The 1, 3 and 5 year survival in patients with FLC was of 66, 40 and 26% respectively. The factors associated with a higher survival in patients with FLC were age more than 23 years, feasibility of surgical resection, free surgical borders, absence of thrombosis or invasion to hepatic vessels and the absence of alterations in liver enzymes. The size of the tumor, gender, cellular proliferation and atypia did not affect the prognosis.

**Conclusion:**

We concluded that FLC patients diagnosed before 23 years of age have worse prognosis than those diagnosed after age 23. Other factors associated with worse prognosis in this study are: lack of surgical treatment, presence of positive surgical margins, vascular invasion, and altered hepatic enzymes.

## Background

Hepatocellular carcinoma (HCC) is the most frequent primary neoplasm of the liver. Its incidence is increasing in western countries mainly due to the growing Hepatitis C Virus epidemics. Though potentially curable with surgery, very few patients are candidates for resection because of tumor extension and poor liver function at diagnosis. The prognosis is poor, with a 1-year survival rate of 22.6% [1].

Fibrolamellar carcinoma (FLC) was described initially in 1958 by Edmonson, as a subtype of HCC [2]. It is characterized by eosinophilic polygonal cells and wide bands of fibrotic tissue settled in parallel laminas surrounded by a clustering of tumor cells [3-6]. It has not been linked with hepatic viruses, alcohol, estrogen use, or other risk factors traditionally associated with HCC. It occurs more frequently in children and adults less than 35 years old. It also exhibits a slower clinical course than the more common HCC. Controversy exists whether FLC has a better prognosis than HCC. Small retrospective series have shown a better survival after resection compared to HCC [4,7-10]. Nevertheless, a recent study of 10 patients with FLC did not report a difference in survival compared to 36 patients with classic HCC [11].

The differences in the prognosis of these patients are probably a consequence of a non cirrhotic liver and the expanding nature tumor growth permits a high rate of surgical resection. In contrast to classic HCC, which has well defined prognostic factors such as Alfa fetoprotein levels, performance status, liver function, and the severity of cirrhosis [12-14], no such predictive standards have been elucidated for FLC. The aim of this study was to describe the clinical, radiographic and histopathologic characteristics of FLC and analyze the factors associated with survival.

## Methods

Medical records of patients with the diagnosis of HCC were reviewed in the Instituto Nacional de Ciencias Médicas y Nutrición Salvador Zubirán from 1990 to 2003. Patients with FLC were then identified from biopsy results. Patients with incomplete medical records or lacking biopsy results were excluded from the study.

Biopsies of patients with FLC were reviewed to confirm the diagnosis; some samples where dyed again to determine histopathologic characteristics such as atypia and mitosis index. Additional samples were analyzed by immunohistochemistry with the avidin-biotin-peroxidase complex and counterstained with hematoxylin; they were incubated overnight at 4°C either with rabbit anti-human proliferation cell nuclear antigen as a marker of cellular synthesis phase. A pathologist of our institution, blinded to the results of survival, counted the number of cell nuclei positive for nuclear antigen found per microscopy field at × 40 magnification in 10 different fields [15].

The variables of survival, alpha fetoprotein, mitotic index and cellular proliferation index were expressed as mean ± standard error. The SPSS program version 10 was used for the statistic analysis. Statistic significance was determined as P < 0.05 with two-sided test. The Student's *t *test and the chi square or Fisher's exact test was used to compare the continuous variables with normal distribution and nominal variables of patients with classic HCC and FLC respectively. Kaplan-Meier curves and log-rank was used for the survival analysis. The multivariate analysis was done using Cox regression modelling.

## Results

**Figure 1:**
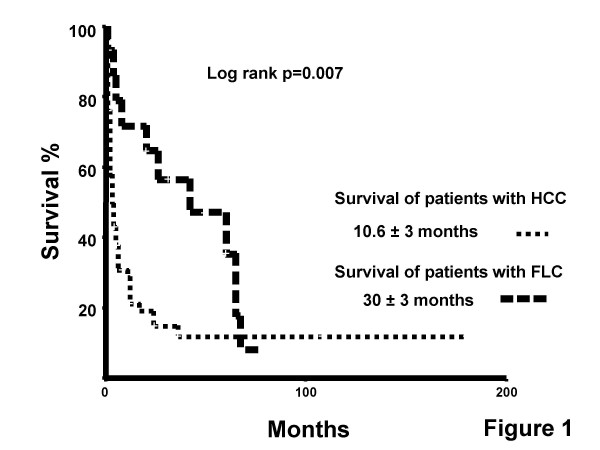
Survival analysis of patients with HCC (n = 159) compared with FLC (n = 15) p = 0.007.

We reviewed 174 medical records of patients with diagnosis of HCC and found 15 patients with FLC,(8.6%). Seventy percent of the FLC patients were females compared to 37% of the classic HCC patients (p = 0.045). The average age was lower in patients with FLC compared to patients with HCC (23 ± 2.6 years with a range of 17–45 years vs 58 ± 1.5 years with a range of 22–87 years respectively p = 0.001). No patient with FLC had a previous history of blood transfusions or a positive serology for hepatitis. In contrast, 75% of patients with HCC had positive hepatitis B and/or C virus serology (p = 0.0001). Thirty percent of the patients with FLC reported use of oral contraceptives, with a range of use of 6–60 months, versus 15% of the patients with classic HCC (p = 0.1). Most common signs and symptoms in patients with FLC were: right upper quadrant pain 83%, hepatomegaly 75%, and weight loss 67%. In laboratory screening we found mean serum albumin of 3.05 ± 0.2 vs 2.9 ± 0.1 g/dL in patients with classic HCC (p = 0.8). The alpha fetoprotein levels were significantly lower in patients with FLC compared to patients with classic HCC (81.5 ± vs 355.7 ± 65 ng/L, p = 0.003). The patients with FLC and classic HCC presented stage I 27% vs 6% (p = 0.026), stage II 7% vs 16% (p = 0.46), stage III 40 vs 67% (p = 0.042) and stage IV 27% vs 10% (p = 0.1) respectively. The mean survival time of patients with FLC was 30 ± 6.4 months vs 10.6 ± 3 of the classic HCC (p = 0.007) (Figure 1).

In FLC, the most frequent radiographic studies done for the diagnosis were CT scan in 92% of the patients, then MRI in 67% and USG in 58%. The most frequent characteristics found on CT scan were: solitary tumor (22% of the patients), lymphadenopathy (14%), linear hypodense images corresponding to central scar (13%), peripheral reinforcement (12%). The MRI characteristics were central scar (40%), defined contour (18%), more than one tumor (12%), and multilobular contour (12%).

**Figure 2:**
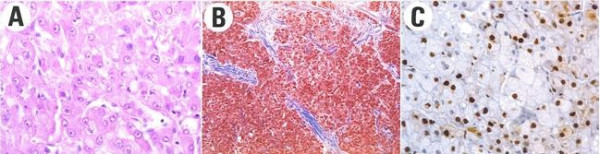
Fibrolamellar Hepatocarcinoma (400#215;), hematoxylin-eosin with big polygonal cells, and abundant eosinophil granular cytoplasm (A). Masson trichromic (100×) with cordons of hepatocytes separated with dense collagen septum (B). Immunohistochemistry for PCNA that shows high index of cellular proliferation of the nucleus (C)

Histopathologic characteristics of FLC biopsies included absence of cirrhosis, presence of fibrotic septum and granular cytoplasm. Furthermore, 92% of the patients demonstrated polygonal cells. Also, 75% of the patients had mild atypia with the remainder showing moderate atypia. In addition, 42% of the biopsies demonstrated pseudoinclusions; 42% had prominent nucleolus, while 33% showed hepatic steatosis (Figures 2A and 2B). The mitotic index average was 2± 1. In all tumors we found a high index of cellular proliferation, mean of 90 ± 5. (Figure 2C).

**Figure 3:**
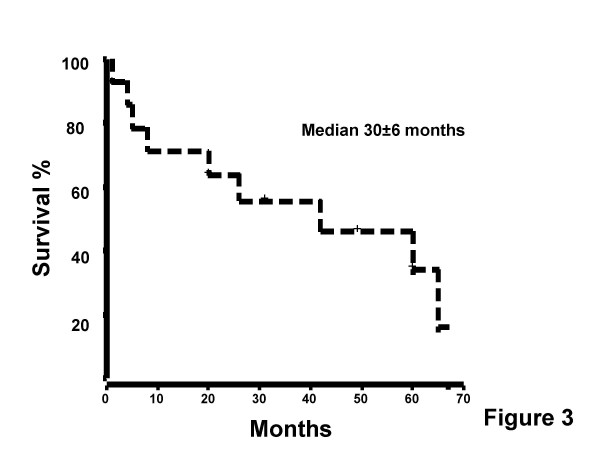
Global survival of patients with FLC at 6, 12, 24, 36 and 60 months was 66, 66, 53, 40 and 26% (median survival of 30 ± 6 months).

**Figure 4:**
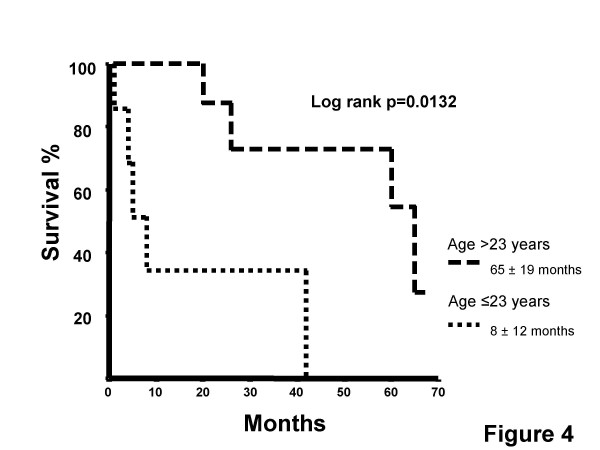
Survival analysis of prognostic factors of patients with FLC. Age = 23 years (n = 7, median survival of 8 ± 2 months) compared to age > 23 years (n = 8, median survival of 65 ± 19 months), p = 0.0132.

**Figure 5:**
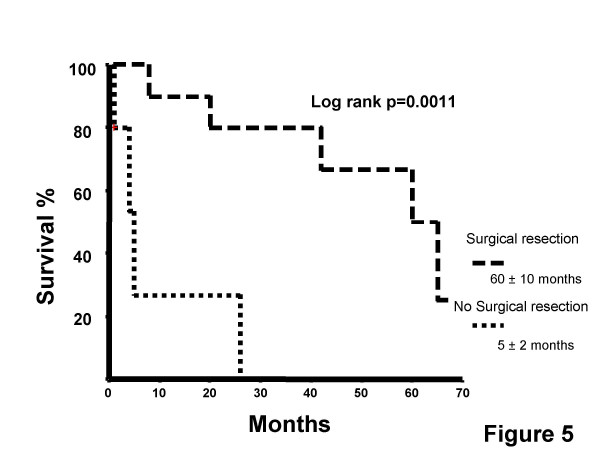
Survival analysis of prognostic factors of patients with FLC. Patients who underwent surgical treatment (n = 10, median survival of 60 ± 10 months) vs patients with medical treatment only (n = 5, median survival of 5 ± 2 months), p = 0.0011.

**Figure 6:**
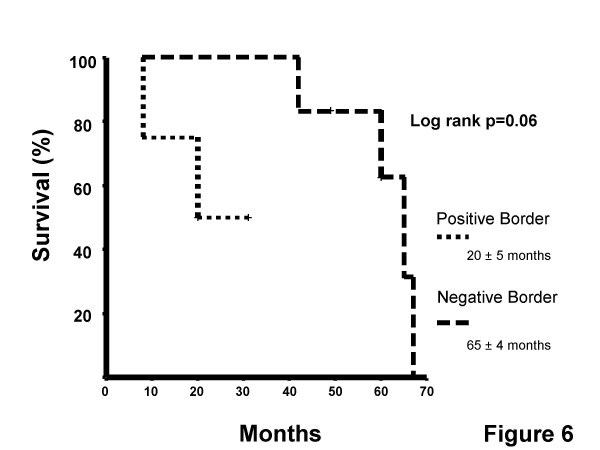
Survival analysis of prognostic factors of patients with FLC. Patients who underwent surgical treatment with positive (n = 5, median survival of 20 ± 5 months) and negative (n = 5, median survival of 65 ± 4 months) surgical borders, p= 0.06.

**Figure 7:**
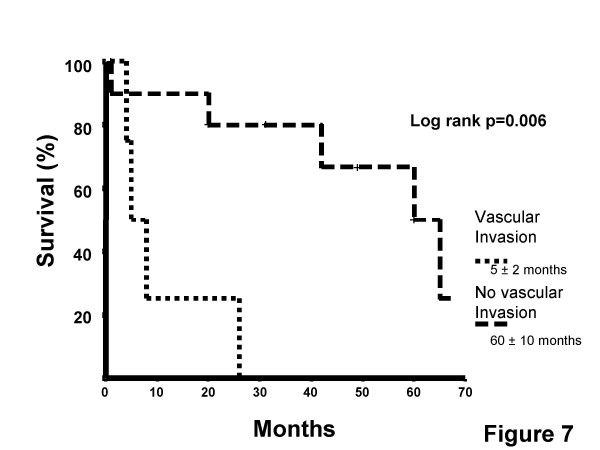
Survival analysis of prognostic factors of patients with FLC. Patients with (n = 9, median survival of 5 ± 2 months) and without (n = 6, median survival of 60 ± 10 months) vascular invasion, p = 0.006.

**Figure 8:**
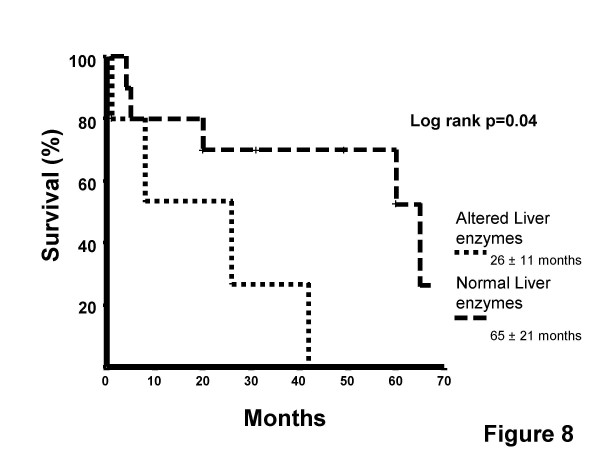
Survival analysis of prognostic factors of patients with FLC. Patients with (n = 10, median survival of 26 ± 11 months) and without (n = 5, median survival of 65 ± 21 months) altered liver enzymes, p = 0.04.

Surgical resection was performed in 80% of the FLC patients. The most frequent procedures were exploratory laparotomy with biopsy (40% of the patients), lobectomy (20%) and segmentectomy (20%). 60% had free surgical margins and 43% percent received chemotherapy. The overall survival of patients with FLC at 6, 12, 24, 36 and 60 months was 66, 66, 53, 40 and 26% (Figure 3. The factors associated with a better survival were age 23 years or more (median of the age) (8 ± 2 vs 65 ± 19 months; p = 0.0132) (Figure 4), resectability (absence of multiple tumors in more than one lobe, absence of involvement of a major branch of portal or hepatic veins and adequate hepatic function) (60 ± 10 vs 5 ± 2 months; p = 0.001) (Figure 5), negative surgical borders for tumor (65 ± 4 vs 20 ± 5 months; p = 0.06) (Figure 6), absence of thrombosis or invasion to the hepatic vein (60 ± 10 vs 5 ± 2 months; p = 0.006) (Figure 7) and normal liver enzymes (ALT and AST) (65 ± 21 vs 26 ± 11 months; p = 0.04) (Figure 8).

Factors unrelated to prognosis were gender (p = 0.646), presence of palpable mass (p = 0.84), hepatomegaly (p = 0.68), weight loss (p = 0.64), ascites (p = 0.57), fever (p = 0.18), jaundice (p = 0.34), hemoglobin value (p = 0.52), normal alkaline phosphatase (p = 0.68), hypoalbuminemia (0.9), increase in alpha fetoprotein (p = 0.67), level of atypia (p = 0.31), mitotic index (p = 0.9), index of cellular proliferation (p = 0.8) and TNM stage (p = 0.69). Multivariate analysis showed only age greater than 23 years (p = 0.027) and absence of multiple tumors in more than one lobe (p = 0.043) were associated with better survival.

## Discussion

Fibrolamellar Hepatocarcinoma is a rare primary hepatic malignancy constituting approximately 1% of all cases of primary liver cancer. A recent retrospective study, the Surveillance, Epidemiology and End Results (SEER) program diagnosed only 68 cases of FLC out 7,896 cases of HCC (0.86%); nevertheless, FLC made up 13.4% of all HCC tumors in people below the age of 40 years [16]. In our study, we found a much higher frequency of 8.6% (15 of 174 patients of all ages). Possibly the number of patients with classic HCC was underestimated in our study as we excluded all patients without biopsies. Almost all patients lacking biopsy were those with advanced cirrhosis, a characteristic of patients with classic HCC rather than FLC. Our study showed a clear absence of association of FLC with Hepatitis virus and the absence of elevation in alpha fetoprotein compared to classic HCC [17].

Another interesting issue was the higher frequency of female gender in the group of FLC compared to classic HCC (60 vs 37%). This observation was also reported in the SEER study where the authors found a higher proportion of females (51.5 vs 26.3%) [16]. Some cases of FLC have been reported during pregnancy [18-20]. In our study, 56% of the female patients with FLC had oral contraceptive use. This was not significantly different from classic HCC patients, but we cannot exclude a hormonal influence in the physiopathology.

We found a significantly lower age at diagnosis in patients with FLC, similar to results reported in the SEER study; the age for FLC at diagnosis was 39 ± 20 vs 65 ± 13. A greater proportion of patients with FLC were classified as having localized disease (stage I) compared to patients with HCC. Similar results were described by the SEER study, where localized disease at diagnosis was seen in 41.2% of FLC patients vs 30.9% of HCC patients. One of the main radiological features found in our study was the presence of central scar. A retrospective study analyzed different radiological features in this kind of tumors and found central scar 71% on CT scans and 82% on MRI in patients with FLC [21].

A limitation of the SEER study was the absence of analysis of factors that modified survival. We found important differences in survival related to age; younger patients (less than 23 years) had a worse prognosis than those who were older. Another associated factor was the feasibility of surgical resection (multiple tumors in more than one lobe) and the presence of tumor in, and thrombosis of, liver vessels independent of the tumor size. We found that histological characteristics such as atypia and cellular proliferation index did not influence prognosis.

The marked differences in epidemiology and clinical course indicate that FLC is distinct from HCC. We found that patients with FLC had higher survival rates than patients with HCC. These results are consistent with the published literature. Some authors report a higher survival rate in patients with FLC after surgical resection, compared to patients with HCC in absence of cirrhosis [9]. In the SEER study they found that the 5 year survival rate was 31.8% for FLC compared with 6.8% for HCC, adjusting for differences in age, gender, race, stage of disease, receipt of resection or transplant, and time of diagnosis. FLC was independently associated with a 46% reduction in risk of mortality [16]. Conversely, another study found no difference in survival following surgical resection between patients with FLC and those with HCC. A multicenter trial of postresection chemotherapy (Pediatric Oncology Study Group Study 8945/Children's Cancer Study Group 8881) compared the survival of 10 children with FLC and 36 with HCC, without finding differences according to histology.

## Conclusion

We concluded that FLC patients diagnosed before 23 years of age have worse prognosis than those diagnosed after age 23. Other factors associated with worse prognosis in this study are: lack of surgical treatment, presence of positive surgical margins, vascular invasion, and altered hepatic enzymes.

## Competing interests

The author(s) declare that they have no competing interests.

## Authors' contributions

JGL and AT reviewed medical records and participated in the design BM reviewed the pathological material and helped to draft the manuscript. MU and ELR participated in the design of the study and coordination. LML participated in the design of the study and performed the statistical analysis. OA conceived of the study, and participated in its design and coordination, performed the statistical analysis and helped to draft the manuscript. All authors read and approved the final manuscript.

## Pre-publication history

The pre-publication history for this paper can be accessed here:



## Figures and Tables

**Table 1:** Association of global survival in patients with FLC to clinical and pathological characteristics.

Variable	Survival (months)	*P *(log rank)
Age		
≤ 23 years	8 ± 2	**0.013**
> 23 years	65 ± 19	
Gender		
male	26 ± 6	0.646
female	42 ± 27	
Surgical		
resection	60 ± 10	**0.011**
No resection	5 ± 2	
Surgical border		
Positive	20 ± 5	**0.06**
Negative	65 ± 4	
Fever		
Present	67 ± 14	0.188
Absent	26 ± 7	
Jaundice		
Present	65 ± 19	0.345
Absent	26 ± 13	
Palpable Mass		
Present	42 ± 33	
Absent	26 ± 14	0.84
Weight loss		
Present	26 ± 6	0.646
Absent	42 ± 27	
Ascites		
Present	4 ± 32	0.578
Absent	42 ± 2	
Hepatomegaly		
Present	42 ± 20	0.681
Absent	20 ± 14	
Vascular Invasion		
Present	5 ± 2	**.0061**
Absent	60 ± 10	
Atypia		
low	26 ± 13	0.319
Moderate	65 ± 9	
Hemoglobin		
≤ 12	26 ± 19	0.527
>12	42 ± 28	
Liver enzymes		
abnormal	26 ± 11	**0.04**
normal	65 ± 21	
Alkaline phosphatase		
abnormal	65 ± 44	0.682
normal	42 ± 17	
Albumin		
abnormal	5 ± 30	0.9
normal	42 ± 19	
Alfa fetoprotein		
abnormal	20 ± 21	0.672
normal	42 ± 21	
TNM		
Stage I and II	65 ± 14	0.693
III	60 ± 12	
IV	8 ± 8	
